# Peripheral Blood Neutrophilia as a Biomarker of Ozone-Induced Pulmonary Inflammation

**DOI:** 10.1371/journal.pone.0081816

**Published:** 2013-12-31

**Authors:** Jenny A. Bosson, Anders Blomberg, Nikolai Stenfors, Ragnberth Helleday, Frank J. Kelly, Annelie F. Behndig, Ian S. Mudway

**Affiliations:** 1 Division of Medicine/Respiratory Medicine and Allergy, Department of Public Health and Clinical Medicine, Umeå University, Umeå, Sweden; 2 Division of Respiratory Medicine and Allergy, Department of Medicine, Ostersund, Sweden; 3 MRC-PHE Centre for Environment and Health, School of Biomedical Sciences, King’s College London, United Kingdom; University of California San Francisco, United States of America

## Abstract

**Background:**

Ozone concentrations are predicted to increase over the next 50 years due to global warming and the increased release of precursor chemicals. It is therefore urgent that good, reliable biomarkers are available to quantify the toxicity of this pollutant gas at the population level. Such a biomarker would need to be easily performed, reproducible, economically viable, and reflective of ongoing pathological processes occurring within the lung.

**Methodology:**

We examined whether blood neutrophilia occurred following a controlled ozone challenge and addressed whether this could serve as a biomarker for ozone-induced airway inflammation. Three separate groups of healthy subjects were exposed to ozone (0.2 ppm, 2h) and filtered air (FA) on two separate occasions. Peripheral blood samples were collected and bronchoscopy with biopsy sampling and lavages was performed at 1.5h post exposures in group 1 (n=13), at 6h in group 2 (n=15) and at 18h in group 3 (n=15). Total and differential cell counts were assessed in blood, bronchial tissue and airway lavages.

**Results:**

In peripheral blood, we observed fewer neutrophils 1.5h after ozone compared with the parallel air exposure (-1.1±1.0x10^9^ cells/L, p<0.01), at 6h neutrophil numbers were increased compared to FA (+1.2±1.3x10^9^ cells/L, p<0.01), and at 18h this response had fully attenuated. Ozone induced a peak in neutrophil numbers at 6h post exposure in all compartments examined, with a positive correlation between the response in blood and bronchial biopsies.

**Conclusions:**

These data demonstrate a systemic neutrophilia in healthy subjects following an acute ozone exposure, which mirrors the inflammatory response in the lung mucosa and lumen. This relationship suggests that blood neutrophilia could be used as a relatively simple functional biomarker for the effect of ozone on the lung.

## Introduction

Epidemiological [1] and field studies [2] have demonstrated that exposure to ambient ozone (O_3_), a major component of photochemical smog, is associated with a wide range of adverse health effects including exacerbations of asthma and COPD [3], as well as the induction of cardiovascular events [4,5]. The underlying mechanisms have been explored in experimental chamber studies in which relatively high concentrations of O_3_ have been shown to elicit a spectrum of acute transient responses, including decrements in lung function, increased airway resistance [6-8], altered airway epithelial permeability [9,10] as well as a spectrum of inflammatory changes characterized by airway neutrophilia [6,8,11-13]. The gold-standard for assessing airway inflammation is based on bronchoscopy with airway lavages and bronchial biopsy sampling. This method is however both invasive and requires a considerable level of technical skill and time to perform. In addition, its invasive nature raises ethical issues regarding sampling at multiple time points in volunteers, which has restricted our understanding of the time course of ozone-induced inflammation in the human lung. To overcome these limitations, numerous groups have championed the use of less invasive techniques, such as exhaled breath condensate [14-16], nasal lavage [17] and induced sputum [18-20]. However, little quantitative association has been found between the magnitudes of response using these methods with bronchoscopy-based lavage. 

The assessment of respiratory and cardiovascular effects in vulnerable populations exposed to pollution has predominately relied on spirometric tests, self-reported symptoms and medical records. At present, research in the field of biomarkers is providing new opportunities with the development of tests to monitor pulmonary inflammation and injury, including the measurement of pneumoproteins [21] and acute phase proteins in blood [22,23], as well as exhaled NO [18] and inflammatory markers in exhaled breath condensate [24] and induced sputum [25]. Surprisingly, to date there are relatively few studies which have examined the differential cell counts in peripheral blood after pollutant exposures, and of these the majority have examined responses to particulate pollution arising from forest fires [26] with only one paper [27] reporting O_3_-induced neutrophilia in the blood.

The aim of the present study was therefore to evaluate whether inflammatory responses in peripheral blood could represent a useful marker for ozone induced airway inflammation. We hypothesized that ozone exposure would induce peripheral blood neutrophilia and that this would be correlated with established biomarkers of ozone-induced inflammation in the airways. 

## Methods

### Subjects

 Healthy non-smoking subjects were exposed to ozone and filtered air using a standardised protocol [28]. All subjects had negative skin prick tests to common allergens and normal lung function. None had a history of airway infection for a period of at least six weeks prior to the exposure, or during the actual study. In the initial study three separate pairs of exposures were performed using the following groups of subjects: In group 1 [n=13, 5 female, 8 male; average age 24.6 years (range 19-31)], bronchoscopy was performed 1.5 hours after the end of the air and ozone exposures. In group 2 [n=15, 9 female, 6 male; 25 years (range 19-32)] at 6 hours and in group 3 [n=15, 5 female, 10 male; mean age 23 years (range 21-27)] 18 hours post exposure. In a follow-up study examining the impact of the subjects sex on the magnitude of the observed ozone-induced inflammation, an additional 14 subjects were exposed under identical conditions, with bronchoscopy performed at the 6 hour post exposure time point to produce a combined group of 29 subjects (16 female, 13 male; 24.5 years (range 19-32)). Subjects were recruited by advertisements and the study was performed with the approval of the local research ethics committee at the University of Umeå, in accordance with the Declaration of Helsinki, and with the written informed consent of all participants. 

### Study design

**Figure 1:**
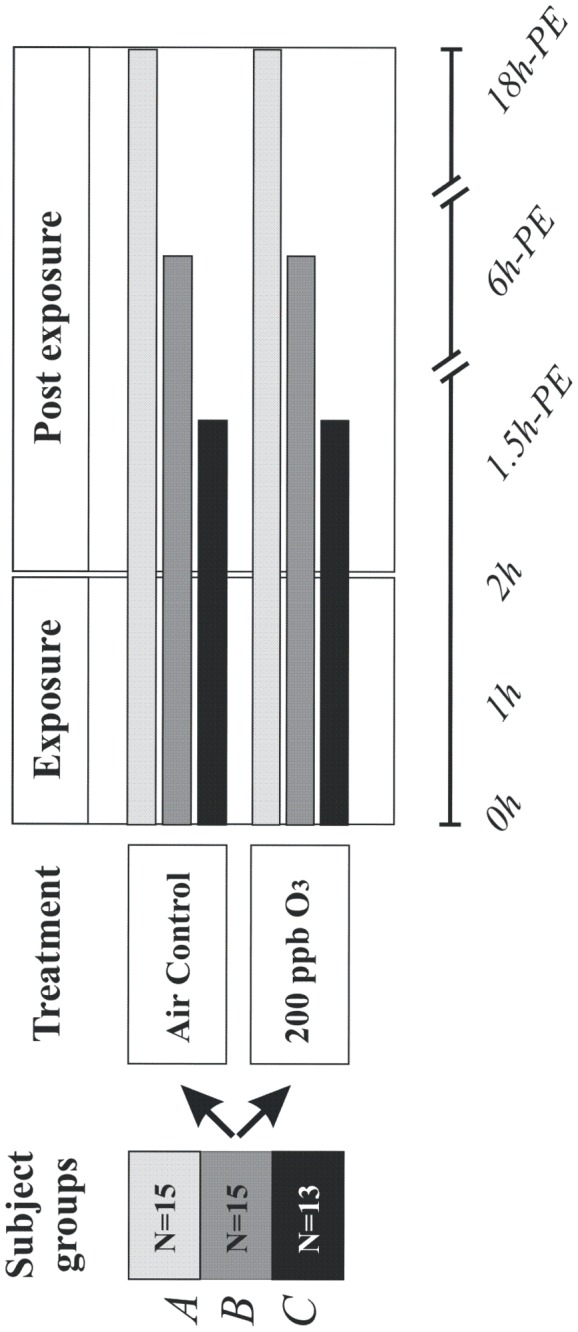
Cartoon of the exposure protocol employed in the current study, with three separate groups undergoing blood and bronchoscopy-based sampling at various times post a standardised two hour filtered air and ozone exposure.

The data on pulmonary inflammation (in BW, BAL and endobronchial mucosal biopsies) in this study are drawn from two previously published ozone challenge studies of healthy subjects exposed to ozone, with bronchoscopy performed at 1.5 [28] and 6 hours [29] post-exposure. In addition, we report here for the first time the results from an equivalent challenge study with airway assessments at 18 hours post-exposure. The data on systemic inflammation from each of these studies has not been previously published. The additional 14 subjects included in the follow-up study are drawn from a previous investigation with airway sampling performed 6 hours post ozone-challenge [30]. Whilst information on inflammatory markers in BW and BAL has been previously published, this represents the first reporting of the biopsy and blood data for this study. Each of the individual studies was performed in a double-blind, crossover manner, with subjects exposed to filtered air and 0.2 ppm of ozone for two hours in an exposure chamber with moderate exercise and rest (Figure 1). The individual challenge studies used in this analysis were therefore performed on different groups of healthy subjects, with the exposures performed in different years. This protocol has previously been described in detail [28]. In the studies cited above, peripheral blood samples were drawn pre-exposure and immediately before the bronchoscopies at 1.5, 6 and 18 hour time points. 

### Ozone exposure

Ozone (O_3_) was generated by a Fischer’s Ozonegenerator 500 MM (Fischer Labor und Verfahrens-Technik, Bonn, Germany), as described in detail previously [28]. 

### Bronchoscopies with lavage and bronchial mucosa biopsy sampling

 Bronchoscopy under local anaesthesia, with bronchial wash (BW; 2 x 20 ml), bronchoalveolar lavage (BAL; 3 x 60 ml) and mucosal biopsy sampling, was performed, as described previously [28]. Briefly, the lavage fluid was filtered and centrifuged and the cell pellet was separated from the supernatant and resuspended in PBS to a concentration of 10^6^ cells/mL. The total number of cells was counted in a Bürker chamber. Cell differential counts were conducted on slides stained with May-Grünwald Giemsa, with 400 cells counted per slide. A FACScan flow cytometer (Becton-Dickinson, Stockholm, Sweden) was used to determine lymphocyte subsets T-cells (CD3+, CD4+ and CD8+) and B-cells (CD19+) in duplicates. BW and BAL fluid IL6 concentrations were determined using an ELISA kit from R&D Systems, Inc. (Minneapolis, MN, USA).

Mucosal biopsies were processed into glycolmethacrylate (GMA) resin and stained using monoclonal antibodies (mAbs) (Table **1**). Quantification of endothelial adhesion molecules in the mucosal blood vessels was carried out by expressing the number of vessels stained with specific mAb as a percentage of the total vessels revealed by staining with the pan-endothelial mAb EN4. The number of cells stained with each mAb was counted separately in the epithelium and in the mucosa. Areas including smooth muscle, glands, large blood vessels, torn or folded tissue within the sections were excluded as were areas more than 100μm beneath the epithelial basal membrane. The length of the epithelium and the area of the mucosa were determined using computer-assisted image analysis (Qwin Colour (RG), Leica Q500MC, Leica, Cambridge, UK). The total number of positive cells was expressed as cells/mm of the epithelium and cells/mm^2^ of the mucosa respectively. 

**Table 1:** Monoclonal antibodies used for immunohistochemistry against inflammatory cells and adhesion molecules.

**Monoclonal Ab**	**Marker**	**Dilution**	**Source**
NE	Elastase, Neutrophils	1:1000	Dako, Glostrup, Denmark
CD 62P	P-selectin, Microvasculature	1:40	Serotec, Oxford, UK
CD 54	ICAM-1, Microvasculature	1:800	Dako, Glostrup, Denmark

### Blood analysis

Analyses of total and differential cell counts were performed using an auto analyser at the Department of Clinical Chemistry, University Hospital, Umeå. A FACScan flow cytometer was used to determine lymphocyte subsets using antibodies against total T-cells with subtypes (CD3+, CD4+ and CD8+) and B-cells (CD19+). 

### Statistics

All statistical analyses were performed using SPSS^®^ version 15.0 for Windows (SPSS Inc., Chicago, IL, USA) Comparison of the post air and ozone neutrophil responses were performed using a paired T-test. Pearson correlation test was used to test correlations between two parameters. A *P*-value < 0.05 was considered significant.

## Results

### Effect of 0.2 ppm ozone on neutrophil numbers in peripheral blood

**Figure 2:**
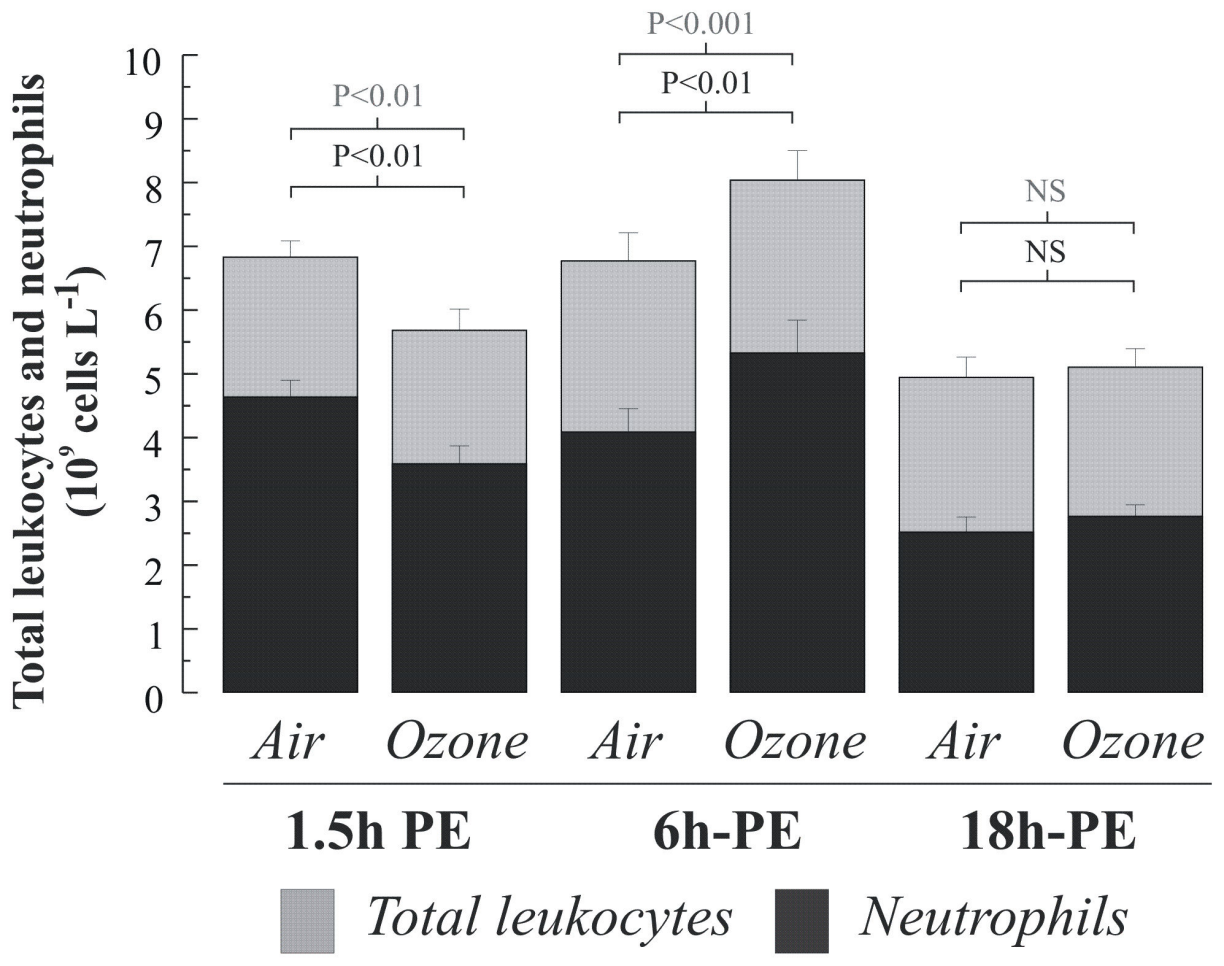
Peripheral blood total leukocyte and neutrophil numbers at 1.5, 6 and 18 hours post air and ozone challenge. Data are illustrated as means with standard deviations. Comparisons of cell numbers post air and ozone challenge were performed using a paired t-test.

Ozone exposure resulted in a decrease in neutrophil numbers in peripheral blood (-1.1x10 ^9^cells/L, p<0.01), which rebounded above filtered air levels at the six hour time point (+1.2 x10^9^ cells/L, p<0.01). By the 18 hour time point, neutrophil numbers did not significantly differ between the ozone and air exposures (Figure **2**). When comparing the neutrophil response following the three air exposures, we found significantly higher neutrophils at the 1.5h and 6h time point versus the 18h time point, indicating an exercise induced neurophilia that had fully attenuated 18 hours after exposure (Figure **2**). No impact of ozone was seen on peripheral blood monocyte, lymphocyte or platelet numbers (Table **2**). Neither was there any change in lymphocyte subsets following ozone (Table **3**). 

**Table 2:** Differential cell counts in peripheral blood sampled from healthy subjects exposed to air and 0.2-ppm of ozone at various times post-exposure.

**Cell type**	**Exposure**	**Sampling time**		
(10^9^ cells L^-1^)		**1.5 h PE**	**6 h PE**	**18 h PE**
**Neutrophils**	**Air**	4.6 ± 0.9	4.1 ± 1.4	2.5 ± 0.9
	**Ozone**	3.6 ± 1.0**	5.3 ± 2.0**	2.8 ± 0.7
**Monocytes**	**Air**	0.4 ± 0.2	0.5 ± 0.1	0.5 ± 0.1
	**Ozone**	0.4 ± 0.2	0.5 ± 0.2	0.5 ± 0.1
**Lymphocytes**	**Air**	1.7 ± 0.3	2.1 ± 0.5	1.7 ± 0.4
	**Ozone**	1.6 ± 0.4	2.1 ± 0.7	1.7 ± 0.4
**Platelets**	**Air**	226 ± 34	250 ± 65	217 ± 46
	**Ozone**	221 ± 41	267 ± 62	221 ± 47

Data are given as means with standard deviation. Comparisons of post air and ozone cell numbers were performed using a paired t-test. ** p<0.01.

**Table 3:** Whole blood lymphocyte subsets in healthy subjects after ozone and air exposure.

**Lymphocyte**	**Exposure**	**Sampling time**		
(%)		**1.5 h PE**	**6 h PE**	**18 h PE**
**CD3+**	**Air**	64.1 ± 21.6	63.2 ± 19.9	68.7 ± 5.7
	**Ozone**	58.3± 29.5	59.7 ± 15.7	66.4 ± 7.6
**CD4+**	**Air**	35.6 ± 11.4	36.5 ± 11.1	41.5 ± 5.9
	**Ozone**	33.2 ± 17.6	34.0 ± 7.8	39.8 ± 7.9
**CD8+**	**Air**	29.1 ± 9.9	26.1 ± 8.7	26.5 ± 6.3
	**Ozone**	24.0 ± 12.9	26.9 ± 7.7	25.3 ± 6.7
**CD19+**	**Air**	5.3 ± 2.4	6.7 ± 7.1	12.1 ± 3.7
	**Ozone**	3.9 ± 2.5	4.7 ± 2.4	10.4 ± 3.4

Data represent are illustrated as means with standard deviation. Comparisons of post air and ozone cell numbers performed using a paired t-test.

### Effect of 0.2 ppm ozone on airway neutrophilia as reflected in bronchial wash, BAL-fluid and bronchial biopsies

**Figure 3:**
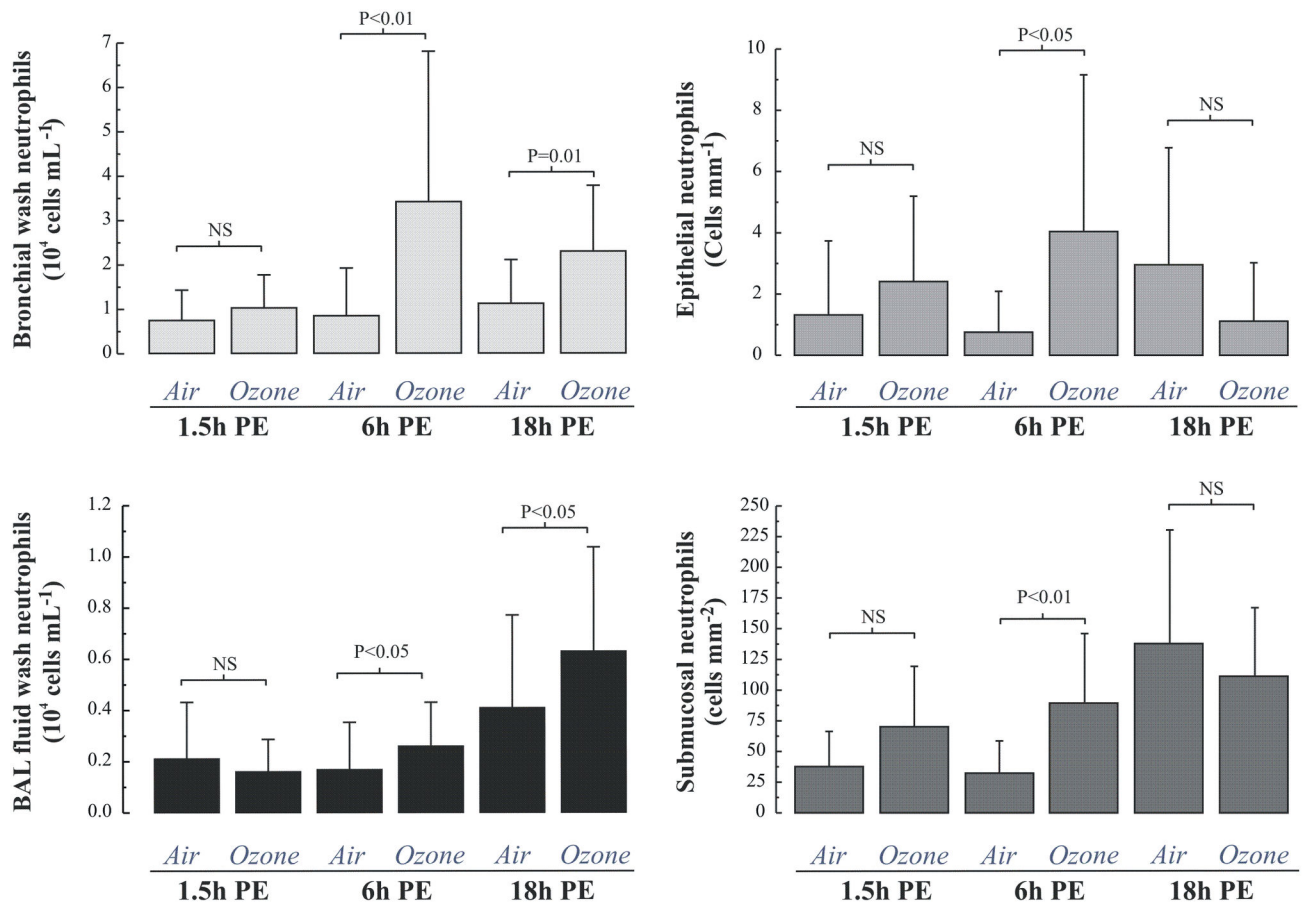
Neutrophil responses in the proximal (bronchial wash) and distal (bronchoalveolar lavage) airway lumen, as well as the epithelium and submucosa of the bronchial airway in healthy subjects exposed to air and ozone. Data are given as means with standard deviation and comparisons of the post air and ozone neutrophil numbers at each time point were performed using a paired t-test.

**Figure 4:**
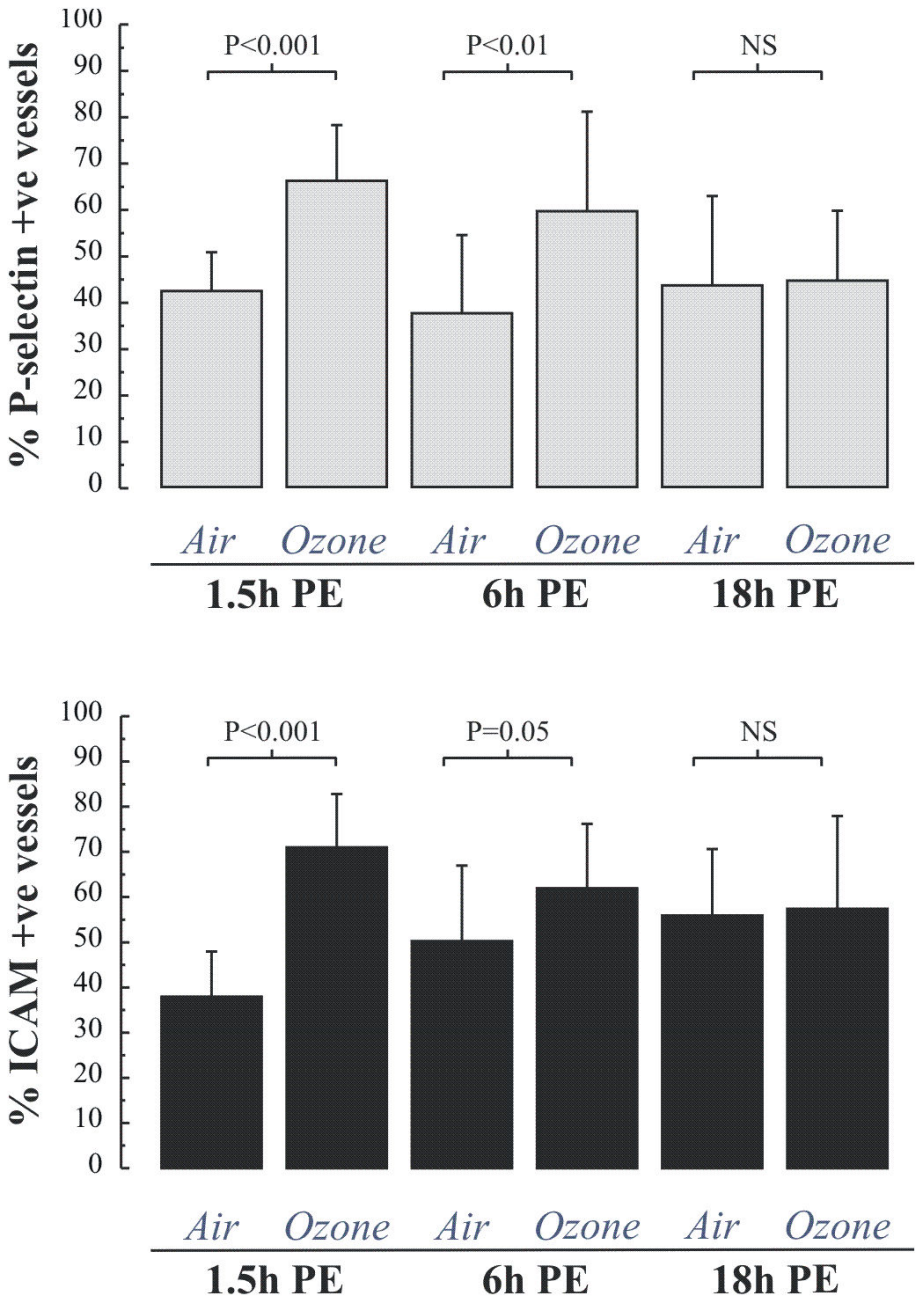
Percentage (mean ± SD) of blood vessels immunostaining for P-selectin and ICAM in the submucosa of bronchial biopsies obtained 1.5, 6 and 18 hours after the end of a 2 hour exposure to air and 0.2 ppm ozone. Comparison of the extent of immunoreactivity was performed using a Students paired t-test.

At 1.5 hours post exposure we saw no evidence of neutrophilia in the BW, BAL or mucosal biopsies (Figure **3**), however an upregulation of vascular endothelial P-selectin (p<0.005) and intercellular adhesion molecule 1 (p<0.005) was observed (Figure **4**). In contrast, a significant neutrophilia was apparent at 6h post-exposure in both the BW (4-fold, p<0.01), BAL-fluid sample (1.5-fold p<0.05), as well as in the bronchial epithelium and submucosa (Figure **3**). At 6 hours post-exposure there was also an up-regulation of P-selectin and ICAM-1 of the same magnitude as seen 1.5h post-exposure (Figure **4**). At 18 hours, the ozone-induced increase in neutrophil numbers persisted both in BW, 1.14±0.98 after air vs. 2.31±1.47 10^4^ cells/mL after ozone (p=0.01), and the more distal compartment sampled by BAL, 0.41±0.36 after air vs. 0.63±0.41 10^4^ cells/mL after ozone, p<0.05 (Figure **3**). In the bronchial submucosa, the neutrophilic inflammation had attenuated at the 18-h time-point, 137±93 after air vs. 111±56 cells/mm^2^ after ozone; p=0.32 (Figure **3**)**.**

### Relationship between ozone-induced neutrophilia in blood, airway lavages and bronchial mucosal tissue

**Figure 5:**
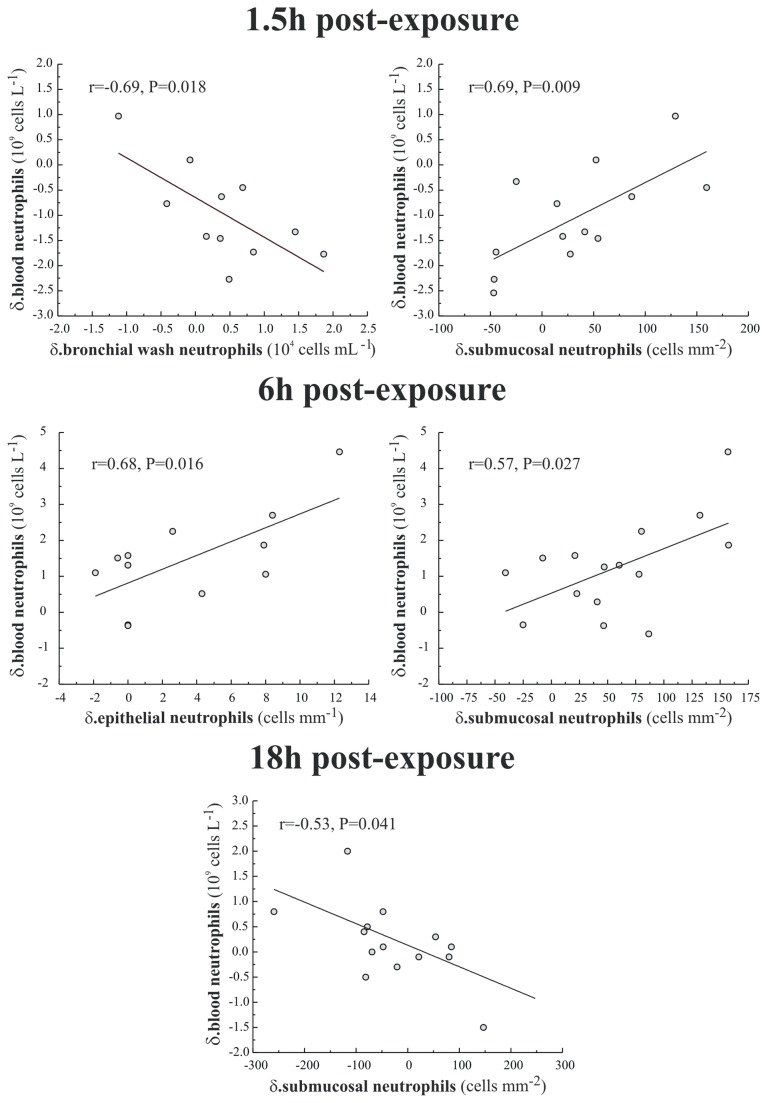
Significant associations (Pearson correlation) between the observed systemic neutrophil response at 1.5, 6 and 18 hour post-exposure (parameter post ozone minus that post air) with that observed in the airway lumen (sampled by BW), the bronchial airway epithelium and submucosa at the equivalent sampling time point. The Pearson correlation coefficient (r) and the 2-tailed significance (P) for each association is illustrated together with a linear regression through the data.

When the neutrophil responses in peripheral blood and bronchial wash were compared, a negative association was detected at 1.5 h post exposure (r=-0.69, p=0.02), suggesting an influx of neutrophils from the blood into the airways, (Figure **5**). At both 1.5 and 6h post exposure there was a positive association between peripheral blood and submucosal neutrophils (r=0.69; p=0.01 and r=0.57, p=0.03), which strengthens the hypothesis that neutrophil counts in peripheral blood reflect the magnitude of pulmonary inflammation. This positive association between blood and submucosal neutrophils was not apparent at the 18 hour time point. 

 Due to the sex imbalance between the three groups, most notably at the critical six-hour time point where blood neutrophilia was apparent, we subsequently augmented this group by an additional 14 subjects to examine possible sex specific differences. In the combined group blood neutrophilia was still apparent 6h post ozone challenge (3.99±1.21 versus 5.17±1.68 x 10^9^ cells L^-1^, p<0.001, with significant increases observed in both male (+1.57±1.17 x10^9^ cells/L, p<0.001, n=13) and female (+0.87±1.36 x10^9^ cells/L, p<0.05, n=16) subjects (Table **4**). The magnitude of ozone-induced neutrophilia did not differ significantly between the males and females in any of the compartments examined. Whilst the evidence of blood neutrophilia was robust at the 6h time point, the relationship with airway inflammation differed markedly by sex. In the female subjects, as in the original group, significant positive correlations were seen with both sub-mucosal (r=0.60, p<0.05) and epithelial neutrophil numbers (r=0.74, p=0.01), with no underlying associations with BW or BAL responses. Conversely, in the male subjects there was no association with the tissue neutrophil response, but positive correlations with p-Selectin and ICAM-1 expression on the vascular endothelium (r=0.82, p<0.05 and r=0.95, P<0.01, respectively) – Table **5**. 

**Table 4:** Markers of the induction of airway and peripheral neutrophilia following the air and ozone challenges in healthy subjects.

		**BW IL6**	**BAL IL6**	**Blood Neut.**	**p-Selectin**	**ICAM-1**	**SM Neut.**	**Epi Neut.**	**BW Neut.**	**BAL Neut.**
		pg mL^-1^	pg mL^-1^	10^9^ cells L^-1^	% +ve vessels	% +ve vessels	Cells mm^-2^	Cell mm^-1^	10^4^ cells mL^-1^	10^4^ cells mL^-1^
	Air	4.28±2.78	1.34±1.19	3.99±1.21	37.44±16.90	50.22±16.75	43.72±27.58	0.71±1.15	1.32±1.28	0.31±0.81
**All**	Ozone	5.05±2.91	2.98±2.36	5.17±1.68	59.49±21.43	61.95±14.25	81.68±48.25	2.19±4.03	3.31±2.60	0.33±0.27
**n=29**	***p-value***	***NS***	**	***	**	***#***	**	***#***	***	***NS***
	δ	0.73±3.63	1.59±2.55	1.19±1.31	22.05±25.40	6.51±16.77	37.96±55.69	1.55±3.80	1.97±2.78	0.01±0.78

	Air	4.46±3.23	1.06±1.13	4.45±1.22	32.73±15.89	46.60±19.44	43.94±29.02	0.79±1.37	1.45±1.41	0.21±0.21
**Female**	Ozone	5.02±3.07	2.85±2.65	5.32±1.94	55.09±21.48	64.01±11.16	77.92±43.53	3.40±5.29	2.64±1.60	0.28±0.28
**n=16**	***p-value***	***NS***	*	*	*	***#***	***#***	***#***	***	***NS***
	δ	0.55±3.62	1.78±2.56	0.87±1.36	22.36±24.08	17.41±23.96	33.99±62.03	2.74±4.78	1.19±1.12	0.07±0.31

	Air	4.05±2.22	1.68±1.22	3.42±0.96	44.50±17.21	55.65±11.06	43.48±27.11	0.62±0.89	1.16±1.13	0.44±1.21
**Male**	Ozone	5.10±2.82	3.15±2.03	4.99±1.45	66.08±21.47	58.85±18.71	85.72±54.38	1.16±2.32	4.21±3.41	0.40±0.26
**n=13**	***p-value***	***NS***	***NS***	***	***NS***	***NS***	*	***NS***	*	***NS***
	δ	0.98±3.80	1.33±2.62	1.57±1.17	21.58±29.64	3.20±14.64	42.24±50.14	0.55±2.49	3.01±3.89	0.07±0.31

Data are presented means with standard deviation for the whole group and separated by sex. The overall response (δ) across the two exposures is also provided. Comparisons of post air and ozone data were performed using a paired t-test, with the overall responses between male and female subjects compared using an unpaired t-test No significant differences were noted between the responses between male and female subjects in any of the endpoints examined.

**Table 5:** Associations between ozone-induced plasma neutrophilia with other markers of neutrophilic inflammation in the lung.

			**Female subjects**								
**Male Subjects**			**Plasma Neut.**	**BW IL6**	**BAL IL6**	**pSelectin**	**ICAM**	**Epi Neuts.**	**SM Neuts.**	**BW Neuts**	**BAL Neuts**
**Plasma Neut.**	Pearson Correlation			-.635**	0.21	0.18	-0.28	.737**	.600*	-0.33	0.20
	Sig. (2-tailed)			0.01	0.43	0.65	0.47	0.01	0.02	0.21	0.46
**BW IL6**	Pearson Correlation		0.27		0.27	-0.27	0.20	-0.60	-0.45	.579*	-0.13
	Sig. (2-tailed)		0.39		0.32	0.49	0.61	0.05	0.11	0.02	0.63
**BAL IL6**	Pearson Correlation		-0.08	0.39		-0.27	-0.36	-0.24	-0.11	0.43	.603*
	Sig. (2-tailed)		0.81	0.21		0.49	0.35	0.48	0.71	0.10	0.01
**pSelectin**	Pearson Correlation		.820*	0.27	0.15		.764*	-0.15	-0.15	-0.03	-0.25
	Sig. (2-tailed)		0.05	0.67	0.81		0.02	0.78	0.69	0.95	0.52
**ICAM**	Pearson Correlation		.945**	0.46	0.04	0.69		-0.29	-0.23	-0.02	-0.15
	Sig. (2-tailed)		0.00	0.44	0.94	0.13		0.57	0.56	0.97	0.70
**Epi Neut.**	Pearson Correlation		-0.07	0.19	-0.14	0.55	0.24		.945**	-0.29	-0.07
	Sig. (2-tailed)		0.83	0.55	0.66	0.26	0.65		0.00	0.40	0.85
**SM Neut.**	Pearson Correlation		-0.03	-0.07	-0.49	0.31	0.06	.699**		-0.23	-0.15
	Sig. (2-tailed)		0.91	0.84	0.11	0.55	0.91	0.01		0.42	0.62
**BW Neut.**	Pearson Correlation		0.41	.610*	-0.05	0.23	0.82	0.48	0.35		0.06
	Sig. (2-tailed)		0.18	0.04	0.87	0.71	0.09	0.12	0.27		0.82
**BAL Neut.**	Pearson Correlation		.635*	0.17	-0.14	-0.17	0.01	-0.01	-0.34	0.30	
	Sig. (2-tailed)		0.03	0.59	0.68	0.79	0.99	0.97	0.28	0.34	

The upper right portion of the matrix presents the associations for the female subjects, with the lower left hand section illustrating the same associations in male volunteers. * P<0.05, ** P<0.01, NS = non significant.

## Discussion

Ozone, a key component of photochemical smog, has been shown to elicit adverse health effects in exposed populations as well as acute toxic responses in the lungs of animals [31] and humans [32] exposed under experimental conditions. Ozone exposure has been associated with retarded lung development [33], incidence of asthma [34], exacerbations of allergic airways disease [35] and increased cardiovascular events [4,5]. Currently, large areas of the US and Europe fail to attain national and international air quality standards for O_3_ [36]. This situation is further compounded by future projections of rising global levels of ozone over the next century related to global warming and increased release of precursor chemicals [36]. Ozone therefore represents one of the major global public health issues of the 21^st^ century.

At the community level, ozone has been shown to affect lung function as well as subjective symptoms and disease severity [32]. However in real world studies there is limited information on airway inflammation, with that available obtained through non-invasive techniques such as exhaled gases [37] and induced sputum [25]. In blood samples some studies have attempted to examine inflammatory status by measuring acute phase proteins and inflammatory mediators [22,23], but the scope of these analyses have been limited due to cost. There is therefore a need to find a reliable, cheap form of biomarker, which can be used to assess pulmonary and systemic inflammation within large cohorts in epidemiological studies. 

In the present study we examined whether ozone induced a systemic inflammation and attempted to relate any observed responses to pulmonary inflammation assessed by means of bronchoscopy lavage and biopsy sampling. For simplicity, we focused on examining differential cell counts in peripheral blood as a biomarker for pulmonary inflammation as this is easily assessed. To date, although numerous groups have examined ozone-induced systemic inflammation in humans by measuring inflammatory mediators and PMN priming, no simple description of the changes in cellularities has been published. After an extensive review of the literature dating back to the original chamber studies by David Bates [38], we have found no quantitative data on this issue beyond a short statement describing peripheral neutrophilia in the paper by Corradi et al [27]. A recent study by Brook et al reported the absence of peripheral blood neutrophilia immediately after, and 24 hours post an experimental exposure to 0.12 ppm of ozone for two hours [39], but this may reflect the low dose and the time point chosen for blood sampling.

In the present study, at a higher ozone dose than employed by Brook et al, ozone was found to elicit a significant blood neutrophilia at 6 hours post-exposure. This was quantitatively related to the inflammatory responses observed within the within the bronchial biospies. At the earlier time point of 1.5h we observed, contrary to our expectations, decreased neutrophil numbers in peripheral blood, which we interpret as reflecting adherence to the vascular endothelium in the lung. Consistent with this view, this response was observed in parallel to increased endothelial expression of p-selectin and ICAM-1, however the responses were not simplistically related quantitatively. At 18 hours post exposure we found no evidence of blood neutrophilia, though inflammation persisted in the airway lumen at this late time point. No other changes in peripheral blood cell types, or lymphocyte subsets were noted at any of the measured time points. The analysis presented is based upon data from three separate inhalation studies and as such, although a standard protocol was employed, we cannot completely exclude the possibility that unknown temporal factors or underlying differences in the studied groups might have confounded the results.

 One issue we were able to address was whether the greater proportion of female subjects at the 6 hour time point influenced the observed blood neutrophilia and its relationship to the parallel inflammatory events occurring within the lung, i.e. whether female subjects were more sensitive to ozone induced inflammation due to their smaller lungs and other gender specific factors. To address this we augmented the original group of 16 subjects at this time point with a further 14 volunteers to obtain sufficient numbers of male and female subjects to perform a meaningful sensitivity analysis. We also examined a panel of markers illustrative of the full pathway of neutrophil migration into the lung from the initial chemotatic signal (IL6), to increased circulating neutrophil numbers, adhesion molecule expression, through to overt tissue neutrophilia within the bronchial epithelium, submucosa and airway lumen (Table **4**). This analysis confirmed the peripheral blood neutrophilia 6 hours post ozone challenge, with equivalent responses in males and females. The study did however illustrate differences in the relationship between this systemic inflammation and the parallel events occurring within the lung. Whilst in the female subjects the systemic neutrophilia was quantitatively related to tissue neutrophil numbers, as per our original analysis, in the males, these associations were absent, with highly significant associations instead observed with the expression of adhesion molecules on the vascular endothelium. One interpretation of this differing relationship is that the neutrophilic response in the lung is more advanced in the females, but our available data does not permit a more informed discussion on this point. Nether-the-less despite the clear sex difference in the relationships observed these data do confirm that the observed systemic neutrophilia is quantitatively related to inflammatory responses occurring in the lung at the time point examined.

This study therefore demonstrates that ozone can induce a significant peripheral neutrophilia at concentrations only 1.7-fold greater than the current European alert threshold (240 µg / m^3^(120 ppb), one hour average,) and raises the possibility that it may have potential as a biomarker for assessing pulmonary inflammation. The time dependence of the response critically highlights the need to sample at, or as close to, the 6-hour post ozone exposure window as is possible. A similar argument is equally valid for all inflammatory and acute phase proteins previously employed to assess inflammation in panel studies [22,23]. As ozone peak concentrations are related to maximal solar radiation this would prioritize sampling in the late afternoon following an ozone episode. 

Clearly, other environmental inhaled xenobiotics are capable of eliciting pulmonary and systemic inflammation and these factors would need to be carefully controlled for in any study attributing inflammation directly to ozone. Notably, exposure to particulate aerosols, both experimentally and in the real-world have provided mixed evidence for the induction of systemic neutrophilia. Exposure of both healthy and sensitive subjects to diesel exhaust PM has provided little evidence of systemic neutrophilia [40], whilst the data arising from concentrated ambient particle studies are inconsistent, with both positive [39] and negative findings [41]. It is therefore possible that different biomarkers of inflammation in the blood may respond differently to ozone and particulate pollution, but this requires further investigation. An additional issue worthy of consideration is how systemic inflammation may vary with consecutive daily exposures to elevated ozone concentrations. This was not addressed in this study, but whilst there is an extensive literature demonstrating attenuation of lung function decrements with multi-day ozone exposures [42], the available data on pulmonary inflammation is mixed, with evidence ranging from apparent attenuation [42], to persistence [43] as well as exacerbation [44]. It therefore remains unclear whether successive ozone challenge induces a chronic persistent inflammation, cycles of equivalent acute episodes, or the induction of tolerance and therefore the relationship between systemic and pulmonary inflammation under these conditions is currently unknown.

In conclusion, we have shown provisional data demonstrating a link between airway and systemic ozone-induced inflammation, using a cost effective, validated and widely available technique. We therefore believe that there is merit in further exploring the relationship between systemic neutrophilia and ozone in the real-world setting.
